# Cancer Care After Natural Disaster: Different Countries, Similar Problems

**DOI:** 10.1200/JGO.19.00155

**Published:** 2019-07-08

**Authors:** Lucilla Verna, Alessio Cortellini, Raffaele Giusti, Corrado Ficorella, Giampiero Porzio

**Affiliations:** Lucilla Verna, MD and Alessio Cortellini, MD, St Salvatore Hospital, and University of L’Aquila, L’Aquila, Italy; Raffaele Giusti, MD, St Andrea University Hospital, Rome, Italy; and Corrado Ficorella, MD and Giampiero Porzio, MD, St Salvatore Hospital, and University of L’Aquila, L’Aquila, Italy

## TO THE EDITOR:

The article by De Guzman and Malik^1^ discusses the treatment of patients with cancer after natural disasters in Asia. The authors point out the additional frailties low- and medium-income countries have to face with in case of natural disasters. They discuss the risks related to the massive population growth in Asia, with the development of “megacities,” where entire neighborhoods arise in at-risk areas within a few years, such as flood-prone areas or high-risk seismic or geological areas. These frailties, of course, together with the socioeconomic vulnerabilities, make these countries even more vulnerable to natural disasters.

Unfortunately, the reported natural disasters have increased worldwide over the decades, until their incidence peaked at the beginning of the 2000s.^2^ This implies that an increasing number of patients with chronic cancer might need medical and social assistance in the future. Our group has already experienced natural disasters; we described the many problems that our home care service handled after an earthquake in 2009.^3^ Indeed, Italy is a country with among the highest risks for seismic and hydrogeological activity, and we have already tried to look for some answers.^4^

According to the World Health Organization’s technical hazard sheet on earthquakes,^5^ the main public health threats vary according to the magnitude of the earthquake, the environment, and the secondary effects of the earthquake. The effects can be summarized in terms of how they relate to immediate health (eg, trauma-related deaths and injuries from building collapse, injuries from the secondary effects of the earthquake) and medium-term impacts on health (eg, increased morbidity and risk of complications of chronic diseases, due to interruption of treatments); the latter is the condition of patients with cancer.

We have appreciated the discussion regarding the critical conditions of patients with cancer during and after natural disasters. De Guzman and Malik^1^ correctly state that disaster management is predominantly a reactive response, and that national health systems still lack proactive attitudes to improve early warning systems, to build resilience, and, most important, to address people with special needs such as patients with cancer.

In our opinion, the lack of preparedness is the biggest problem. National health systems of high-income countries are totally unprepared to face natural disasters. Preparedness of health care professionals is a hot topic in the field; a recent survey conducted among anesthesiologists revealed that few receive sufficient education and training in disaster medicine.^6^ Similar results were also reported among Australian surgeons.^7^ Even if cancer care professionals and oncologists are not the first clinicians called to face natural disasters and large-scale emergencies, they probably are crucial in later phases, when restoring the continuity of care becomes a priority. A recent systematic review by Man et al^8^ summarized the problems that patients with cancer and health care professional have to face after a disaster: the health care infrastructures, the health care workforce, patient relocation, and data dispersion.

In 2015, the United Nations member states adopted the Sendai Framework for Disaster Risk Reduction: 2015–2030,^9^ which clearly states that in case of a disaster, patients with chronic conditions are considered in policy and plans, ensuring they have access to lifesaving services. The problems regarding management of patients with chronic illness, and patients with cancer, in particular, are the same for low-, middle-, and high-income countries. The question is: Why we are determined to plan and prevent only when the nature forces us to face these problems? Enhanced disaster preparedness must be a shared goal of institutions and scientific societies worldwide. The optimal goal would be to prepare specialized medical response teams for immediate and medium-term health impact. Communities are often the first responders, so we think it is crucial to invest as well in the preparedness of general practitioners and all cancer care professionals.
